# Serum CIRP increases the risk of acute kidney injury after cardiac surgery

**DOI:** 10.3389/fmed.2023.1258622

**Published:** 2024-01-03

**Authors:** Zhe Feng, Xiantong Cao, Changying Zhao, Jialan Niu, Yang Yan, Tao Shi, Junjun Hao, Xinglong Zheng

**Affiliations:** Department of Cardiovascular Surgery, The First Affiliated Hospital of Xi’an Jiaotong University, Xi'an, Shaanxi, China

**Keywords:** cardiac surgery, cardiopulmonary bypass, acute kidney injury, cold-induced RNA-binding protein, risk factor

## Abstract

**Introduction:**

Acute kidney injury (AKI) is a frequent perioperative complication. The underlying mechanisms of cardiac surgery-associated AKI are still not completely elucidated. Cold-induced RNA-binding protein (CIRP) has been subsequently found to be regulated by various stress conditions. During cardiac surgery and cardiopulmonary bypass (CPB), the host is subjected to hypothermia and inadequate organ perfusion, resulting in an upregulation of CIRP secretion. The aim of this study is to evaluate the role of elevated extracellular CIRP level as a contributing factor in the development of AKI.

**Methods:**

A total of 292 patients who underwent cardiac surgery were retrospectively enrolled and their serum samples were collected preoperative and postoperative. Demographic data, intraoperative data, in-hospital outcomes, and the occurrence of AKI were also collected for the patients. The correlation between CIRP and intraoperative procedures, as well as its association with postoperative outcomes were analyzed.

**Results:**

In multivariable analysis, higher ΔCIRP (*p* = 0.036) and body mass index (*p* = 0.015) were independent risk factors for postoperative AKI. Meanwhile, patients with postoperative AKI exhibited lower survival rate in 2-year follow-up (*p* = 0.008). Compared to off-pump coronary artery bypass grafting surgery, patients who underwent on-pump coronary artery bypass grafting, valve surgery, aortic dissection and other surgery showed higher ΔCIRP, measuring 1,093, 666, 914 and 258 pg/mL, respectively (*p* < 0.001). The levels of ΔCIRP were significantly higher in patients who underwent CPB compared to those who did not (793.0 ± 648.7 vs. 149.5 ± 289.1 pg/mL, *p* < 0.001). Correlation analysis revealed a positive correlation between ΔCIRP levels and the duration of CPB (*r* = 0.502, *p* < 0.001). Patients with higher CIRP levels are at greater risk of postoperative AKI (OR: 1.67, *p* = 0.032), especially the stage 2–3 AKI (OR: 2.11, *p* = 0.037).

**Conclusion:**

CIRP secretion increases with prolonged CPB time after cardiac surgery, and CIRP secretion is positively correlated with the duration of CPB. Cardiac surgeries with CPB exhibited significantly higher levels of CIRP compared to non-CPB surgeries. Elevation of CIRP level is an independent risk factor for the incidence of AKI, especially the severe AKI, and were associated with adverse in-hospital outcomes.

## 1 Introduction

Cardiac surgery induces activation of various cells within the circulatory system, leading to the secretion of multiple cytokines, chemokines, reactive oxygen species, and other inflammatory factors. These inflammatory factors can trigger various postoperative complications (1–3). Meanwhile most cardiac surgeries require the use of cardiopulmonary bypass (CPB). During the CPB process, there is a significant increase in the expression of inflammatory mediators such as interleukins (IL), tumor necrosis factor-alpha (TNF-α), and kinins, including bradykinin, by the leukocytes (4–6). This exacerbates the inflammatory response during the cardiac surgery and leads to severe complications (7–9).

The occurrence rate of acute kidney injury (AKI) following cardiac surgery, which is a frequent perioperative complication, ranges between 5 and 42% (10–12). It associated with higher mortality rates, prolonged hospital stays, and increased healthcare expenses (12, 13). The underlying mechanisms of cardiac surgery-associated acute kidney injury (CSA-AKI) are still not completely elucidated, and it is a complex condition likely influenced by multiple factors, which may include hypoperfusion, ischemia–reperfusion injury, neurohumoral activation, inflammation, oxidative stress, nephrotoxins, and mechanical factors (12).

Cold-induced RNA-binding protein (CIRP), belonging to the cold shock protein family, is the first cold shock protein discovered in mammalian cells (14). It has been subsequently found to be regulated by various stress conditions, including hypoxia, UV radiation, glucose deprivation, heat stress, H_2_O_2_ (15, 16). This indicates that CIRP functions as a broad stress-response protein. In-depth investigation of CIRP has revealed that its functionality depends on the protein’s distribution location, exerting its effects through both intracellular and extracellular forms. Intracellular CIRP translocates from the nucleus to the cytoplasm, where it binds to and stabilizes various messenger RNAs (mRNAs) within the cell, thereby promoting translation to cope with various external stressors, such as hypoxia, hypothermia and oxidative stress (17–19). Extracellular CIRP has been identified as a novel inflammatory mediator that can stimulate the release of pro-inflammatory cytokines from microglia/macrophages, thereby exacerbating organ damage (17, 20, 21). During cardiac surgery and CPB, the host is subjected to hypothermia and inadequate organ perfusion, resulting in an upregulation of CIRP secretion. In animal experiments, it has been demonstrated that mice with knocked-out CIRP gene exhibited milder renal inflammation and lower levels of inflammatory markers (22). Ischemia–reperfusion injury and CIRP release may occur after the body receives pulsating blood perfusion (23).

This study enrolled patients who underwent cardiac surgery and detected their serum CIPR levels before and after surgery, aiming to evaluate whether the elevated level of extracellular CIRP is a risk factor for the postoperative development of AKI.

## 2 Methods

### 2.1 Patients

A total of 292 patients who underwent cardiac surgery in the First Affiliated Hospital of Xi’an Jiaotong University from January 2018 to December 2019 were retrospectively enrolled, including 249 patients experienced CPB, and 43 patients without it. All patients were followed up until February 2022 with a maximum of 48 months. The primary outcomes were defined as either mortality or loss of follow-up. The study was approved by the Ethics Committee of the First Affiliated Hospital of Xi’an Jiaotong University (Approval No. XJTU1AF2021LSL-028), and informed consent was obtained from all patients.

### 2.2 Clinical data collection

The baseline data included age, sex, body mass index (BMI), smoking history, hypertension, diabetes mellitus, prior myocardial infarction, unstable angina, cardiac functional classification of New York Heart Association (NYHA), left ventricular ejection fraction, preoperative sCr (μmol/L), and preoperative estimated glomerular filtration rate (eGFR, mL/min) were obtained.

The operative data consisted of the duration of cardiopulmonary bypass (CPB) and the duration of aortic cross-clamp. The types of surgeries were also recorded including off-pump coronary artery bypass grafting (OPCAB), on-pump coronary artery bypass grafting (ONCAB), valve surgery, aortic dissection surgery (Sun’s procedure) and other surgeries such as atrial or ventricular septal defect repair or atrial myxoma resection.

In-hospital outcome data included the incidence of AKI, the length of stay in the intensive care unit (ICU), the length of postoperative hospital stay, the mechanical ventilation time, the utilization of postoperative continuous renal replacement therapy (CRRT), SOFA score, and in-hospital mortality. AKI occurred within 7 days after surgery was defined according to the Kidney Disease Improving Global Outcomes Definition and Staging criteria as an absolute rise of ≥26 μmol/L or 0.3 mg/dL within 48 h or 0.3 mg/dL within 48 h after surgery or urine output<0.5 mL/kg/h for more than 6 h or 50–99% Cr (sCr) rise from baseline compared with preoperative baseline values. We did not accurately count the postoperative urine volume due to the retrospective analysis.

### 2.3 Serum data collection

Peripheral blood samples were collected from each patient preoperative and postoperative, then centrifuged and stored at −80°C. The concentration of plasma CIRP was determined using a commercially available ELISA kit (Cusabio, Wuhan, China), following standardized instructions. The CIRP after surgery minus the CIRP after anesthesia was defined as ΔCIRP.

### 2.4 Statistical analysis

Continuous variables were presented as mean ± standard deviation or medians with interquartile ranges, while categorical variables were expressed as percentages. The t-test was used to compare normally distributed continuous data, and the nonparametric Mann–Whitney’s U test was used for skewed data. The chi-square test was used to compare categorical data. Pearson’s correlation analysis was conducted to evaluate the relationships between ΔCIRP and CPB time or cross-clamp time. Univariate logistic regression analysis was performed to identify demographic factors, preoperative laboratory examination results, and operative data factors significantly associated with AKI in all patients. Variables with a value of *p* < 0.05 in the univariate logistic regression analysis were included in the multivariate logistic regression analysis.

All statistical analyses were conducted with a two-sided approach, and a value of *p* <0.05 was deemed statistically significant. The data were analyzed using IBM SPSS software, version 26.0 (IBM Corp., New York, New York, United States).

## 3 Results

### 3.1 Baseline characteristics

The demographic features of the 292 individuals who received cardiac surgery are presented in Table 1. The patients had a median age of 53 (42, 61) years, with 65% being male. The average preoperative creatinine and estimated glomerular filtration rate (eGFR) were 63 (53, 74) μmol/L and 103 (94, 117) mL/min/1.73m^2^, respectively. Among the patients, 118 (40.4%) experienced AKI after the cardiac surgery. The elderly (*p* = 0.039), higher BMI (*p* < 0.001), hypertension (*p* = 0.040), higher EURO Score (*p* = 0.001), higher eGFR (*p* = 0.048), longer CPB time (*p* < 0.001), longer cross clamp time (*p* = 0.003) and higher ΔCIRP (*p* = 0.007) patients were more likely to undergo AKI than those who did not develop AKI.

**Table 1:** The baseline characteristics of the patients enrolled in the study.

Variables	All patients, *n* = 292	AKI, *n* = 118	No AKI, *n* = 174	*p*-value
Age (years)^*^	53 (42, 61)	55 (46, 64)	52 (38,61)	0.039
Male (%)	65	66	64	0.76
BMI (Kg/m^2^)^*^	23 (21, 25)	24 (21, 26)	22 (20, 25)	0.001
Smoking history (%)	46	45	47	0.467
Hypertension (%)	36	43	31	0.040
Prior myocardial infarction (%)	16	15	16	0.831
Unstable angina (%)	20	22	19	0.458
NYHA III/IV (%)	57	56	57	0.870
LVEF (%)^*^	62 (53, 68)	61 (52, 68)	62 (54, 68)	0.707
Preoperative creatinine (μmol/L)^*^	63 (53, 74)	64 (52, 74)	62 (54, 75)	0.620
Cleveland clinic score	3 (3, 4)	3 (3, 4)	3 (3, 4)	0.235
EURO Score	3 (1, 6)	4.5 (2, 8)	2 (0, 5)	0.001
CRP (mg/L)	45 (26, 67)	44 (30, 73)	44 (25, 61)	0.065
Preoperative eGFR (mL/min/1.73m^2^)^*^	103 (94, 117)	101 (92, 113)	105 (96, 120)	0.048
CPB time (min)^*^	90 (52, 121)	103 (71, 129)	82 (39, 117)	0.001
Cross clamp time (min)^*^	58 (11, 81)	65 (43, 84)	49 (0, 77)	0.003
Minimum temperature during CPB (°C)	31 (30, 33)	31 (30, 32)	31 (30, 35)	0.001
ΔCIRP (pg/mL)^*^	693 (216, 1,003)	816 (343, 1,125)	610 (126, 907)	0.007

BMI, body mass index; NYHA, New York Heart Association; LVEF, left ventricular ejection fraction; eGFR, estimated glomerular filtration rate; CPB, cardiopulmonary bypass; CIRP, cold inducible RNA-binding protein.

*Variables are presented as median (25th, 75th percentiles).

### 3.2 Multivariable analysis

In multivariable analysis, BMI (OR: 1.097, 95% confidence interval [*CI*]: 1.018–1.182, *p* = 0.015), higher ΔCIRP (above the median value, OR: 1.724, 95% *CI*: 1.035–2.873, *p* = 0.036) were independent risk factors for postoperative AKI (Table 2).

**Table 2:** Multivariable predictors of acute kidney injury after cardiac surgery.

Variables	OR (95% CI)	*p*-value
Age (years)	1.005 (0.990, 1.021)	0.502
BMI (Kg/m^2^)	1.097 (1.018, 1.182)	0.015
Hypertension	1.224 (0.705, 2.124)	0.472
Preoperative eGFR (mL/min/1.73m^2^) ^*^	0.995 (0.984, 1.006)	0.402
Higher ΔCIRP	1.724 (1.035, 2.873)	0.036

BMI, body mass index; eGFR, estimated glomerular filtration rate; CIRP, cold inducible RNA-binding protein. OR, odds ratio.

*Variables are presented as median (25th, 75th percentiles).

### 3.3 Survival analysis

**Figure 1:**
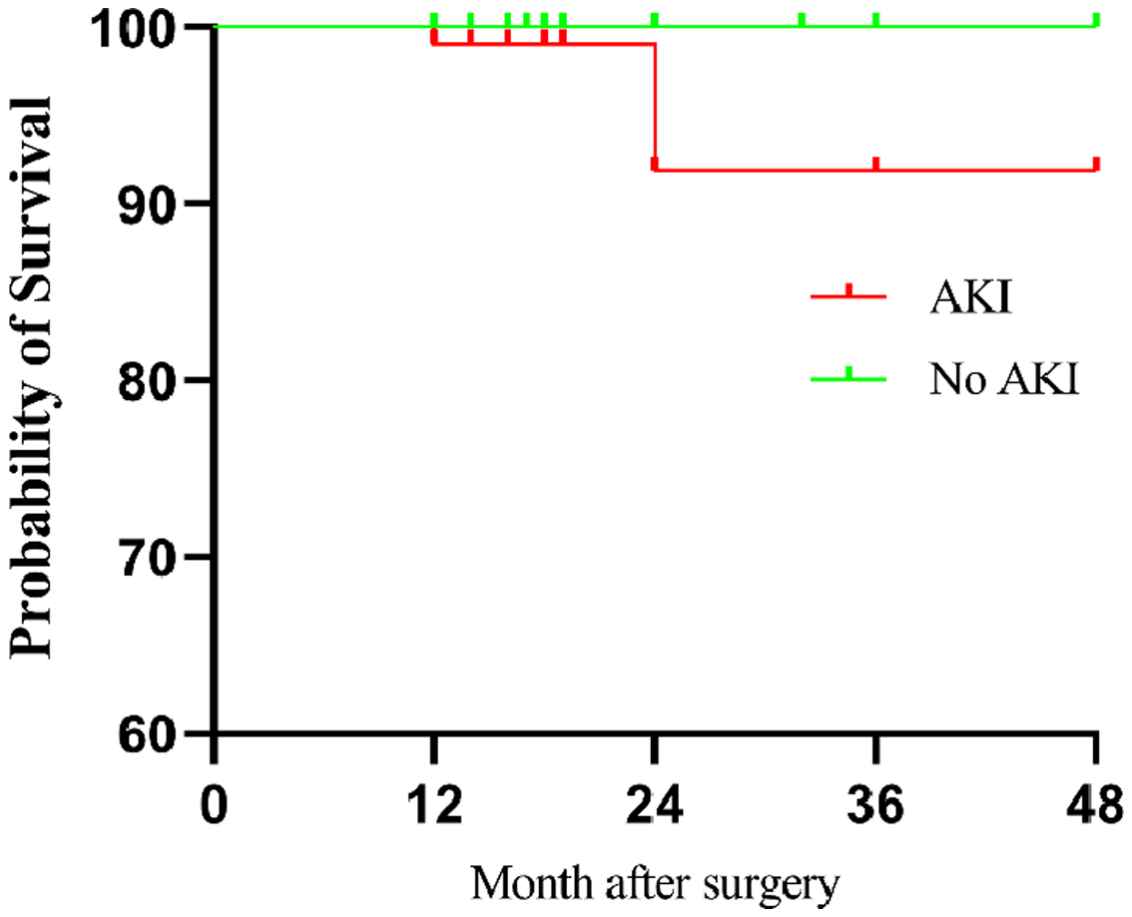
Survival curve of patients with or without AKI after cardiac surgery.

During the follow-up, patients who developed AKI postoperatively exhibited significantly lower survival rate compared to patients who did not experience AKI (*p* = 0.008, Figure 1).

### 3.4 Surgery types and AKI

**Figure 2:**
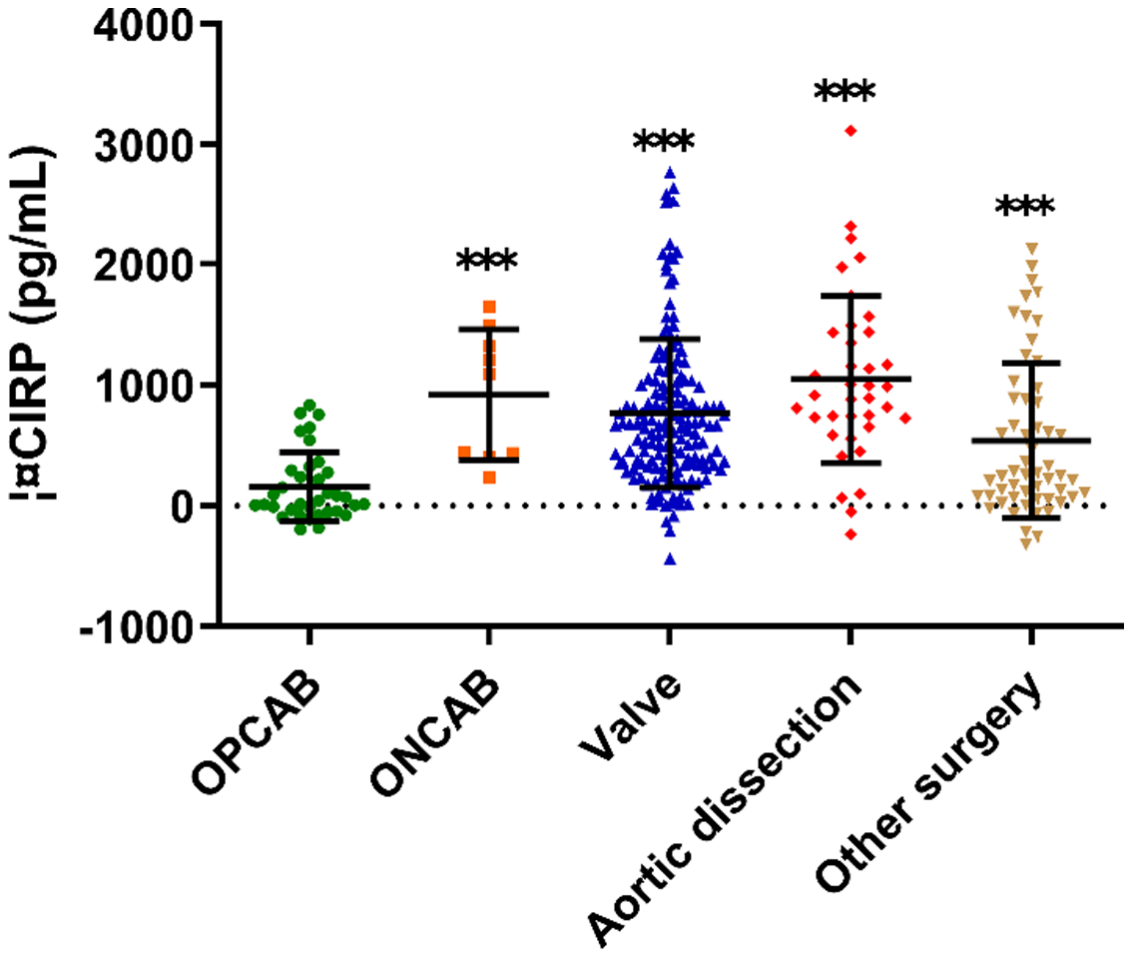
The ΔCIRP for different type of cardiac surgery. CIRP, cold-inducible RNA-binding protein; OPCAB, off-pump coronary artery bypass grafting; ONCAB, on-pump coronary artery bypass grafting.

In the OPCAB group, the average of ΔCIRP was 44 pg./mL (Figure 2), and the incidence of AKI was 31.4%, which was at a low level (Table 3). Compared to OPCAB group, patients who underwent ONCAB, valve surgery, aortic dissection and other surgery had higher ΔCIRP, measuring 1,093, 666, 914, and 258 pg./mL, respectively (*p* < 0.001, Figure 2). The incidence of AKI of four groups were 33.3%, 42.3, 67.6, and 23.6%, respectively (Table 3).

**Table 3:** The type of cardiac surgery.

Variables	OPCAB	ONCAB	Valve	Aortic dissection	Other surgery
No. of patients	35	9	156	37	55
CPB time (min)^*^	−	120 (82, 122)	100 (82, 123)	137 (109, 158)	49 (33, 65)
Cross clamp time (min)^*^	−	66 (13, 92)	70 (53, 87)	77 (61, 88)	0 (0, 37)
Cystatin C (mg/L)	1.5 (1.2, 1.8)	1.6 (1.4, 1.7)	1.5 (1.2, 2.0)	2.4 (1.7, 3.3)	1.1 (0.9, 1.4)
AKI (%)	31.4	33.3	42.3	67.6	23.6
ΔCIRP (pg/mL)^*^	44 (−38, 284)	1,093 (434, 1,320)	666 (349, 1,034)	914 (729, 1,437)	258 (78, 884)

OPCAB, off-pump coronary artery bypass grafting; ONCAB, on-pump coronary artery bypass grafting; CPB, cardiopulmonary bypass; AKI, acute kidney injury.

*Variables are presented as median (25th, 75th percentiles).

### 3.5 CPB time and AKI

**Figure 3:**
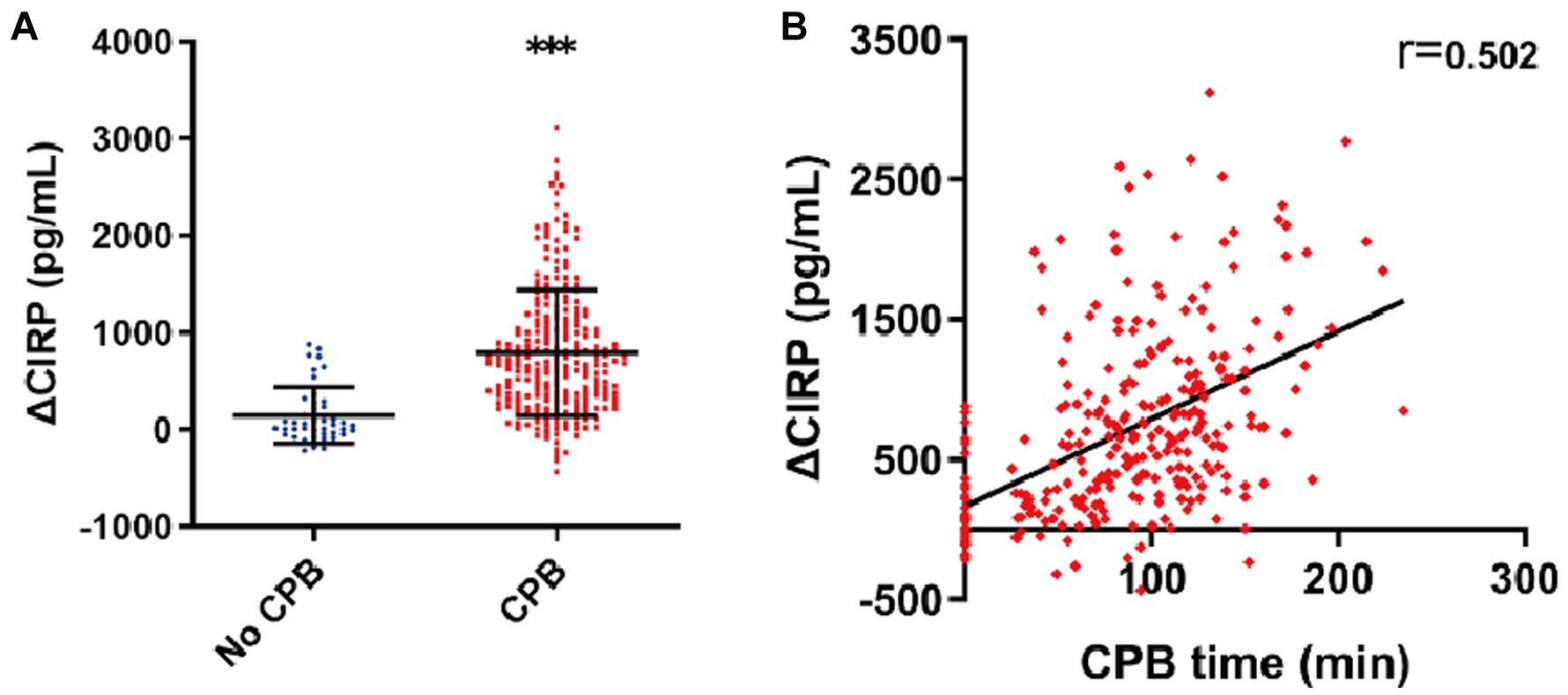
Elevated CIRP after CPB. **(A)** The ΔCIRP levels in patients who experienced CPB were 4.3-fold higher than those who did not. **(B)** The ΔCIRP levels were positively correlated with the CPB time (*n* = 292, *r* = 0.502, *p* < 0.001).

A total of 249 patients underwent CPB during the surgery, while 43 patients did not. The levels of ΔCIRP were significantly higher in patients who underwent CPB compared to those who did not (793.0 ± 648.7 vs. 149.5 ± 289.1 pg./mL, *p* < 0.001, as depicted in Figure 3A). Correlation analysis revealed a positive correlation between ΔCIRP levels and the duration of CPB (*r* = 0.502, *p* < 0.001, as shown in Figure 3B).

### 3.6 ΔCIRP level and AKI

In accordance with the median value of ΔCIRP (593 pg./mL), the patients were categorized into two groups: the high-expression group and the low-expression group. The baseline of two groups were shown in Table 4. Patients with higher ΔCIRP have a higher incidence of unstable angina (25 vs. 14%, *p* = 0.020), longer CPB time (109 vs. 67 min, *p* < 0.001) and cross clamp time (70 vs. 39 min, *p* < 0.001), and lower minimum temperature during CPB (32 vs. 31°C, *p* < 0.001).

**Table 4:** Baseline characteristics of the patients with different ΔCIRP level.

Variables	All patients (*n* = 292)	High ΔCIRP (*n* = 146)	Low ΔCIRP (*n* = 146)	*P*-value
Age (years)^*^	53 (42, 61)	54 (41, 63)	53 (43, 61)	0.836
Male (%)	65	69	61	0.141
BMI (Kg/m^2^)^*^	23 (21, 25)	23 (21, 25)	23 (21, 26)	0.880
Ever smoked (%)	46	51	41	0.17
Hypertension (%)	36	33	39	0.271
Prior myocardial infarction (%)	16	19	13	0.208
Unstable angina (%)	20	25	14	0.02
NYHA III/IV (%)	57	58	55	0.555
LVEF (%)^*^	62 (53, 68)	63 (53, 67)	61 (53, 68)	0.717
Preoperative creatinine (μmol/L)^*^	63 (53, 74)	63 (54, 74)	63 (52, 76)	0.867
Preoperative eGFR (mL/min/1.73m^2^)^*^	103 (94, 117)	103 (94, 117)	103 (93, 115)	0.666
Euro SCORE^*^	3 (1, 6)	2 (1, 5)	3 (1, 7)	0.067
CPB time (min)^*^	90 (52, 121)	109 (81, 133)	67 (0, 100)	<0.001
Cross clamp time (min)^*^	58 (11, 81)	70 (49, 91)	39 (0, 66)	<0.001
Minimum temperature during CPB (°C)	31 (30, 33)	31 (30, 31)	32 (30, 37)	<0.001

CIRP, cold inducible RNA-binding protein; BMI, body mass index; NYHA, New York Heart Association; LVEF, left ventricular ejection fraction; eGFR, estimated glomerular filtration rate; CPB, cardiopulmonary bypass.

*Variables are presented as median (25th, 75th percentiles).

Patients with higher levels of ΔCIRP exhibited a greater incidence of postoperative acute kidney injury (AKI) in comparison to those with lower ΔCIRP levels (Table 5). The incidence of AKI was 47% in the high ΔCIRP group, whereas it was 34% in the low ΔCIRP group, resulting in an OR of 1.67 (95% *CI*, 1.04–2.68, *p* = 0.032). Specifically, patients with high ΔCIRP levels demonstrated a significantly higher occurrence of severe AKI (stage 2 or stage 3) (17 vs. 9%, *p* = 0.037, OR (2.11 [1.04, 4.32])). In the group with higher ΔCIRP level, patients had a higher level of cystatin C (1.6 vs. 1.4 mg/L, *p* = 0.017), longer mechanical ventilation (21 vs. 16 h, *p* < 0.001), prolonged ICU stays (3d vs. 2d, *p* = 0.003), extended hospital stays (11d vs. 10d, *p* < 0.001) and had higher SOFA scores (4.4 vs. 3.5, *p* < 0.001). Additionally, the incidence of acute lung injury was higher in the higher ΔCIRP group (44 vs. 32%, *p* = 0.045, OR (1.62 [1.01, 2.62])).

**Table 5:** In-hospital outcomes of patients with different ΔCIRP level.

Variables	Low ΔCIRP	High ΔCIRP	*p*-value	OR
AKI (%)	34	47	0.032	1.67 (1.04, 2.68)
AKI 1 stage	25	30	0.431	
AKI 2–3 stage	9	17	0.037	2.11 (1.04, 4.32)
Cystatin C (mg/L)^*^	1.4 (1.1, 1.9)	1.6 (1.3, 2.2)	0.017	−
Mechanical ventilation (h)^*^	16 (7, 23)	21 (16, 32)	<0.001	−
ALI (%)	32	44	0.045	1.62 (1.01, 2.62)
ICU stay (d)^*^	2 (1, 4)	3 (2,5)	0.003	-
Postoperative hospital stay (d)^*^	10 (7, 13)	11 (8, 19)	<0.001	−
Postoperative CRRT use (%)	2.7	4.8	0.363	1.78 (0.51, 6.20)
In-hospital mortality (%)	1.4	4.8	0.09	3.63 (0.74, 17.76)
SOFA Score^*^	3.5 (2, 5)	4.4 (3, 5)	<0.001	−

AKI, Acute kidney injury; ALI, acute lung injury; ICU, intensive care unit; CRRT, continuous renal replacement therapy; SOFA, sequential organ failure assessment; OR, odds ratio.

*Variables are presented as median (25th, 75th percentiles).

## 4 Discussion

AKI is a severe postoperative complication following cardiac surgery, leading to increased postoperative ICU stay, prolonged total hospitalization duration, and decreased postoperative survival rates. CIRP secretion increases with prolonged CPB time after cardiac surgery, and its secretion level is positively correlated with the duration of CPB. Cardiac surgeries requiring longer CPB durations exhibit significantly higher levels of CIRP compared to non-CPB surgeries. Elevation of CIRP levels is an independent risk factor for the occurrence of AKI and can exacerbate the severity of AKI and lead to poorer in-hospital outcomes for patients. Therefore, CIRP can serve as a predictive marker for postoperative AKI and in-hospital outcomes in cardiovascular surgery.

A meta-analysis has pointed out several risk factors for CSA-AKI. These include the presence of pulmonary hypertension, cyanotic heart disease, univentricular heart, administration of nephrotoxic drugs, use of vasopressors, CPB time, reoperation, low preoperative creatinine, high preoperative eGFR and sepsis (24). The pathophysiological mechanisms of CSA-AKI are not fully understood, but currently, they mainly involve nephrotoxins, ischemia and reperfusion, cardiac dysfunction, venous congestion, inflammation, and oxidative stress (25). In recent years, there has been increasing recognition that the systemic inflammatory response syndrome observed in response to CPB is characterized by widespread manifestations throughout the body (1). It has become evident that individual patients exhibit significant heterogeneity in the extent to which distinct inflammatory pathways are activated. However, CIRP secreted by various organs during cardiac surgery is also an important factor that cannot be ignored. In the univariate analysis of this study, the levels of CIRP were significantly higher in the AKI group compared to the non-AKI group. In the multivariate analysis, elevated levels of CIRP were identified as an independent risk factor for AKI. The mice experiment conducted by Siskind et al. confirmed that CIRP can induce acute kidney injury by activating renal TREM-1 (26, 27). Moreover, a prospective observational study conducted by Xia et al. demonstrated that post-CPB levels of CIRP are associated with an increased risk of pulmonary dysfunction. CIRP can serve as one of the indicators for predicting postoperative lung injury (28). So, CIRP may serve as a marker of organ damage after heart surgery.

CIRP is an intracellular stress-response protein and a specific type of damage-associated molecular pattern (DAMP). Wang et al. provided evidence showing that Extracellular CIRP (eCIRP) can act as a DAMP, initiating the activation of innate Toll-like receptor 4 (TLR4)/MD2-mediated pro-inflammatory signaling, which subsequently induces inflammation (29). Furthermore, studies have indicated that eCIRP can modulate the promotion of macrophages and neutrophils in immune responses and inflammation through the TREM-1 signaling pathway. (30, 31).It exhibits the ability to respond to diverse stress stimuli by modulating its expression and mRNA stability. In this study, we found that patients with high levels of CIRP experienced longer CPB times and lower CPB temperatures. It was also found in this study that patients with higher CIRP levels had a higher incidence of ALI and a higher SOFA score. This is consistent with the research conclusion of Xia et al. (28). During cardiac arrest and CPB, organs are under conditions of non-pulsatile blood flow, inadequate perfusion, and exposure to hypothermic stimuli, all of which can lead to significant secretion of CIRP by the body. Extracellular CIRP (eCIRP) is associated with a range of conditions and can activate immune and inflammatory responses (32). After the cardiac arrest, the organ experiences ischemia reperfusion injury, and a large amount of CIRP will aggravate the ischemia reperfusion injury and further aggravate the organ function impairment (33, 34). Patients with higher CIRP level take longer time to recover. In this study, it was found that patients with higher CIRP levels experience longer mechanical ventilation time, higher cystatin C levels and incidence of ALI, longer ICU stay and hospital stay. During the follow-up, we did not find that a high level of CIRP would affect the long-term survival rate of patients, which may be related to the fact that CIRP is mainly a stress-response protein, which mainly increases during and after the operation and gradually returns to normal level within 3 days after the operation (28), which is not enough to cause long-term postoperative changes, but in this study, we found that it can predict the short-term postoperative outcome. Research has also found that inhibiting the expression or function of CIRP can attenuate the severity organ damage (22, 34, 35). Targeting CIRP with pharmacological or genetic interventions may reduce inflammation, alleviate renal tissue damage, and improve kidney function. Despite the association between CIRP and postoperative AKI, research in this field is still in its early stages, and further studies and validations are needed to elucidate the specific mechanisms and treatment strategies. Therefore, in clinical practice, more research and validation are required for CIRP-targeted intervention therapies to determine their potential role in postoperative acute kidney injury following cardiac surgery.

There are certain limitations in this study. We employed the median method to distinguish high and low levels of CIRP, which, due to the sample size, has limited clinical significance. In the future, larger sample sizes are needed to establish more precise cutoff values. Additionally, the mechanisms by which CIRP mediates acute kidney injury have not been clearly elucidated in this article, and current available evidence does not establish a causal relationship between the two, warranting further investigation.

## 5 Conclusion

CIRP levels were positively correlated with CPB time during the cardiac surgery. This study demonstrated that patients with elevated level of CIRP before and after cardiac surgery are more prone to experiencing poorer in-hospital outcomes and developing AKI, especially severe AKI. The elevated levels of CIRP may be an independent risk factor for AKI and were associated with adverse in-hospital outcomes.

## Data availability statement

The raw data supporting the conclusions of this article will be made available by the authors, without undue reservation.

## Ethics statement

The study was approved by the Ethics Committee of the First Affiliated Hospital of Xi’an Jiaotong University (Approval No. XJTU1AF2021LSL-028), and informed consent was obtained from all patients.

## Author contributions

ZF: Data curation, Formal analysis, Investigation, Methodology, Software, Writing – original draft. XC: Data curation, Methodology, Writing – review & editing. CZ: Data curation, Methodology, Writing – review & editing. JN: Data curation, Methodology, Writing – review & editing. YY: Methodology, Supervision, Writing – review & editing. TS: Supervision, Data curation, Writing – review & editing. JH: Supervision, Methodology, Writing – review & editing. XZ: Supervision, Conceptualization, Funding acquisition, Project administration, Resources, Writing – review & editing.
